# Differential mRNA Expression Profiling Reveals the Role of MiR-375 in Inflammation of Bovine Mammary Epithelial Cells

**DOI:** 10.3390/ani12111431

**Published:** 2022-06-01

**Authors:** Yuhang Li, Qichao Hu, Zhuoma Luoreng, Jian Yang, Xingping Wang, Yun Ma, Dawei Wei

**Affiliations:** 1 School of Agriculture, Ningxia University, Yinchuan 750021, China; 12020131317@stu.nxu.edu.cn (Y.L.); hqc13629560035@126.com (Q.H.); yangjian9603@163.com (J.Y.); mayun@nxu.edu.cn (Y.M.); weidaweiwdw@163.com (D.W.); 2 Key Laboratory of Ruminant Molecular Cell Breeding, Ningxia Hui Autonomous Region, Yinchuan 750021, China

**Keywords:** miR-375, dairy cow, mastitis, RNA-seq, bMEC, MAPK signaling pathway

## Abstract

**Simple Summary:**

Bovine mammary epithelial cells (bMECs) are often used as cell models for mammary gland research. They are the most important cells for mammary gland function and the first line of defense for pathogen identification. MicroRNAs (miRNAs) are important regulatory factors involved in many physiological and pathological processes. Here, we examined a transcriptome profile of bovine mammary epithelial cell lines transfected with miR-375 inhibitor or negative control (NC) inhibitor, and further reveal the potential role of miR-375 in bMECs by differentially expressed mRNA analysis. We found that miR-375 potentially promotes inflammation in the mammary gland through the MAPK signaling pathway.

**Abstract:**

MicroRNAs (miRNAs) are a class of small non-coding RNAs that regulate post-transcriptional gene expression and several biological processes. Bovine mammary epithelial cells (bMECs) mediate critical immune responses in the mammary gland and the occurrence of mastitis. Current research focuses on miRNA regulation of bMECs, but the miR-375 regulatory mechanism in bMECs is unclear. This study explored the role of miR-375 by profiling the transcriptome of miR-375-silenced bMECs using RNA-seq and identifying differentially expressed mRNAs (DIE-mRNAs). There were 63 DIE-mRNAs, including 48 down-regulated and 15 up-regulated mRNAs between miR-375-silenced bMECs and the controls. The Kyoto encyclopedia of genes and genomes (KEGG) and Gene Ontology (GO) functional analysis showed that the DIE-mRNAs enriched nuclear receptor subfamily 4 group A member 1 (*NR4A1*) and protein tyrosine phosphatase non-receptor type 5 (*PTPN5*) anti-inflammatory genes of the mitogen-activated protein kinase (MAPK) signaling pathway. However, they showed an opposite trend to the expression of miR-375 silencing, suggesting that miR-375 promotes bMEC inflammation through the MAPK signaling pathway. The findings of this study provide a new reference for understanding the regulation of bMEC inflammation and cow mastitis.

## 1. Introduction

Mastitis causes significant economic losses to the agricultural sector by reducing milk production and quality [1]. Many factors affect mastitis, including the genetics of the animal, which influences the susceptibility or resistance of the animal to the disease [2]. The cow mammary gland primarily comprises the bovine mammary epithelial cells (bMECs) involved in the synthesis and secretion of milk [3]. Furthermore, it is the first barrier preventing pathogens from invading cow mammary glands by secreting several immune regulatory factors [4]. Therefore, bMECs are important in the milk production and mammary gland immunity of dairy cows. However, many genes regulate the growth activity and function of bMECs [5,6,7].

MicroRNAs (miRNAs) are a class of single-stranded endogenous non-coding small RNA molecules with 20–25 nucleotides [8]. They negatively regulate mRNA transcription and translation by targeting the 3’UTR of messenger RNAs (mRNAs), thereby regulating various cellular activities, including proliferation, differentiation, development, apoptosis, inflammation, and other biological processes [8,9]. Recently, numerous studies examined the differential expression of miRNAs in bovine mammary epithelial cells [10,11]. For example, miRNA-145 regulates the expression of immune cytokines in bovine with mastitis by targeting the expression of the fascin actin-bundling protein 1 (*FSCN1*) gene [12]. MiR-125b [13] and miR-146a [14] aggravate and alleviate mastitis in dairy cows by targeting the NF-κB inhibitor-interacting Ras-like 2 (NKIRAS2) and Toll-like receptor 4 (TLR4)/tumor necrosis factor receptor-associated factor 6 (TRAF6)/NF-κB pathways, respectively. In addition, in lipoteichoic acid (LTA)-induced inflammation of bMECs, miR-23a inhibits the inflammatory response by directly targeting PI3K [15]. MiR-204-5p promotes lipid synthesis in mammary epithelial cells by targeting sirtuin 1 (SIRT1) [16]. Despite these reports, the regulatory patterns and mechanisms of numerous miRNAs remain unclear.

MiR-375, a member of the miRNA family, was first described in MIN6 and TC1 cells of the mouse pancreas. MiR-375 is involved in islet formation and insulin secretion [17]. In recent years, studies have revealed that human miR-375 regulates various physiological and pathological functions of cells. For instance, miR-375 regulates the occurrence and development of colorectal cancer, knee osteoarthritis, acinar cells inflammation, and nasopharyngeal carcinoma by targeting the phosphatidylinositol-4,5-bisphosphate 3-kinase catalytic subunit alpha (*PIK3CA*), autophagy-related 2B (*ATG2B*), autophagy-related 7 (*ATG7*), and pyruvate dehydrogenase kinase 1 (*PDK1*) genes, respectively [18,19,20,21]. Additionally, human miR-375 participates in breast cancer progression [22]. MiR-375 is one of the most down-regulated miRNAs in breast tissues following *Staphylococcus aureus* and *Escherichia coli* infection, suggesting that it regulates the immune response and inflammation in the mammary gland [23]. However, whether and how miR-375 regulates inflammation in the mammary gland are not well understood. The mammary alveolar cells (MAC-T) cell line is the conventional model for studying the molecular regulation of mammary gland function in dairy cows [24,25]. The transcriptome profiles of miR-375-silenced MAC-T were analyzed using the RNA-seq technique to understand how miR-375 regulates the expression of genes in bMECs and, by extension, the mammary gland.

## 2. Materials and Methods

### 2.1. Cell Culture

MAC-T cell lines identified and frozen in our laboratory were used in this research [24,26]. The cells were thawed and cultured in DMEM/F12 (Hyclone, Logan, UT, USA) medium supplemented with 10% fetal bovine serum (System Biosciences, Mountain View, CA, USA) at 37 °C under 5% CO_2_ and 100% humidity.

### 2.2. Transient Transfection of MiR-375 Inhibitor

MAC-T cells were seeded and cultured in 6-well plates. When the cells reached 60–70% confluence, they were transiently transfected with miR-375 inhibitor or negative control (NC) (final concentration 100 nM) using the X-Tremegene HP DNA Transfection Reagent (Roche, Basel, Switzerland), according to the manufacturer’s instructions. The experiment was performed in triplicate (3 samples in the inhibitor NC group and 3 samples in the miR-375 inhibitor group). The transfection efficiency was detected by observing the red fluorescence of Cy3-labeled inhibitor NC under an inverted fluorescence microscope (Olympus Corporation, Tokyo, Japan), and the expression detection inhibition efficiency of miR-375 was analyzed by real-time quantitative PCR (qPCR). The miR-375 inhibitor (5′-UCACGCGAGCCGAACGAACAAAA-3′) and NC (5′-CAGUACUUUUGUGUAGUACAAA-3′) were designed and synthesized by RiboBio Co., Ltd. (Guangzhou, China).

### 2.3. Sample Collection and RNA Extraction

The MAC-T cells were collected 48 h after transfection. Total RNA was extracted from the transfected MAC-T using the TriZol kit (Takara Biomedical Technology Co., Ltd., Beijing, China) following the manufacturer’s protocol. The concentration and quality of RNA were determined using agarose gel electrophoresis and a Multi-Mode Reader (BioTek, Winooski, VT, USA) (Table S1). High-quality RNA samples were used to construct cDNA libraries as described below.

### 2.4. Constructing cDNA Libraries and High-Throughput Sequencing

The sequencing library was constructed using the ABclonal mRNA-seq Lib Prep Kit (ABclonal, Wuhan, China), following the manufacturer’s protocol. The mRNA was enriched by magnetic beads with Oligo (dT). The mRNA was randomly interrupted by the fragmentation and converted to double-stranded cDNA. Then, the double-stranded cDNA was purified using AMPure XP beads (Beckman Coulter, Pasadena, CA, USA). Further, the purified double-stranded cDNA was end repaired, A-tail enriched, and connected to sequencing joints. Then, AMPure XP Beads were used for fragment size selection. The selected fragments were PCR enriched and used to obtain the final cDNA library. Agilent Bioanalyzer 4150 (Agilent Technologies, Palo Alto, CA, USA) evaluated the library before qPCR validation. Then, the high-throughput sequencing was performed using the Illumina Novaseq 6000 (Illumina, San Diego, CA, USA) platform in Shanghai Applied Protein Technology (Shanghai, China).

### 2.5. Data Processing and Quality Control

After high-throughput sequencing, the raw data were stored in the FASTQ file format. We conducted an overall assessment of the quality of the raw reads by Perl, including the sequencing error rate, ATGC content, raw data composition, and comparative analysis with the reference genome to ensure the reliability of the sequencing results. Clean reads were obtained by removing the connector sequences and filtering out low-quality reads (the number of bases with a base mass value ≤25 accounted for over 60% of the total reads). The proportion of N (undetermined bases) was <5%. The obtained clean data were mapped to the bovine reference genome UMD 3.1 (http://oct2018.archive.ensembl.org/Bos_taurus/Info/Index (accessed on 25 March 2021)) using HiSAT2 software (http://daehwankimlab.github.io/hisat2/ (accessed on 25 March 2021)). Finally, clean reads were used for subsequent analysis.

### 2.6. Analysis of Differentially Expressed mRNAs

The expression levels of each gene in each sample, expressed as Fragments Per Kilobase of transcript sequence per Million base pairs sequenced (FPKM), were calculated using *FeatureCounts* (http://subread.sourceforge.net/ (accessed on 26 March 2021)). Pearson’s correlation analysis was performed between each sample. DESeq2 (http://bioconductor.org/packages/release/bioc/html/DESeq2.html (accessed on 26 March 2021)) software was used to analyze the differentially expressed mRNA in the miR-375 inhibitor group and the inhibitor NC group. The significant differentially expressed mRNAs were identified following the *p*-value < 0.05 and |log_2_^FoldChange^| > 1 threshold.

### 2.7. qPCR

We inquired the sequence information of miR-375, designed a neck ring structure primer specific for reverse transcription of miR-375 (5′-GTCGTATCCAGTGCAGGGTCCGAGGTATTCGCACTGGATACGACTCACGCGA-3′) and conducted reverse transcription reaction of miR-375. In addition, eight differentially expressed mRNAs (DIE-mRNAs) (3 up-regulated and 5 down-regulated mRNAs) were randomly selected for validating the RNA-seq results. Briefly, the 1 µg total RNA in bMECs was reverse transcribed into cDNA using the PrimeScriptTM RT Reagent Kit with gDNA Eraser (Takara Biomedical Technology Co., Ltd., Beijing, China) following the manufacturer’s instructions. The 2×M5 HiPer SYBR Premix EsTaq (withTli RNaseH) fluorescence quantitative detection kit (Mei5 Biotechnology Co., Ltd., Beijing, China) detected the expression of DIE-mRNAs in transfected MAC-T cells using a CFX-96 Touch Real-Time PCR instrument (BioRad, Hercules, CA, USA). The qPCR reaction mix included 10.0 μL of 2×M5 HiPer SYBR Premix EsTaq (with Tli RNaseH), 0.8 μL of 10 nmol/μL forward and reverse primers, 100 ng of cDNA template, and adding sterilized deionized water to 20.0 μL. The reaction conditions included initial denaturation at 95 °C for 30 s, subsequent 40 cycles of denaturation at 95 °C for 5 s, and annealing at 60 °C for 30 s. All the experiments were repeated three times. The qPCR primers are listed in Table 1. All qPCR experimentation and analysis were performed following the minimum information for publication of quantitative real-time PCR experiments (MIQE) guidelines [27]. The amplification efficiency of the primers are 95–105%.

The data were analyzed using SPSS software version 25.0 using *GAPDH* and *RPS18* [28] as internal controls. The relative expression levels of the genes were calculated using the 2^−ΔΔCt^ method [29] and expressed as the mean ± standard deviation (X ± SD). The SPSS 25.0 software was used to test the significance of differential expression between groups through independent-samples *t*-tests, and *p* < 0.05 was considered statistically significant.

### 2.8. Functional Annotation of DIE-mRNAs

Gene cluster analysis was performed to display the gene expression pattern in different samples visually. Gene Ontology (GO) analysis for the molecular function (MF), biological process (BP), and cellular component (CC) of the DIE-mRNAs was performed using the clusterProfiler package in the R software (version: 4.0.3). Kyoto encyclopedia of genes and genomes (KEGG) analysis was also performed to identify pathways and the biological function of genes regulated by miR-375.

## 3. Results

### 3.1. The Silencing Efficiency of MiR-375 in MAC-T

**Figure 1:**
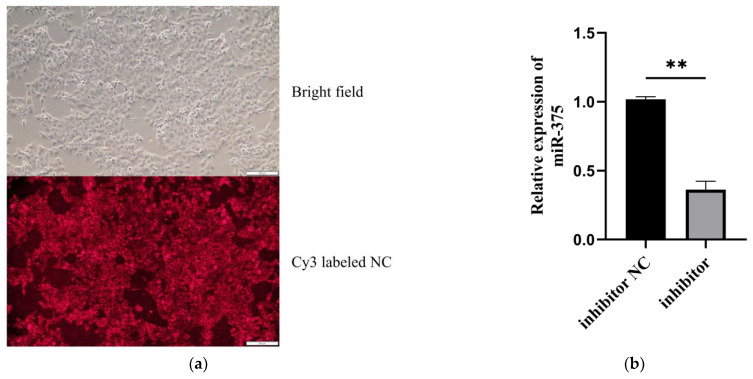
The miR-375 silencing efficiency in MAC-T. (**a**) Cy3-labeled inhibitor NC transfected into the cells detected using an inverted fluorescence microscope. (**b**) Analysis of miR-375 silencing efficiency in MAC-T using qPCR, ** *p* < 0.01.

Inverted fluorescence microscope revealed that miR-375 in MAC-T cells was efficiently silenced after transfection with the corresponding inhibitor (Figure 1a), consistent with qPCR findings (*p* < 0.01) (Figure 1b). The results showed that the silencing effect of miR-375 in MAC-T cells was good, and subsequent experiments could be carried out.

### 3.2. Sequence Data Analysis

We sequenced cDNA libraries using the Illumina high-throughput sequencing platform to study the miR-375 inhibitory effect on bMECs. The sequence data from the experimental and NC groups are listed in Table 2. The Q30 was over 92.5%, indicating a high base quality. Moreover, high-quality reads were obtained after removing the joint and low-quality reads (Table 2). In general, these findings demonstrated the accuracy and reliability of the sequence data.

### 3.3. Overall Distribution of mRNA Expression

**Figure 2:**
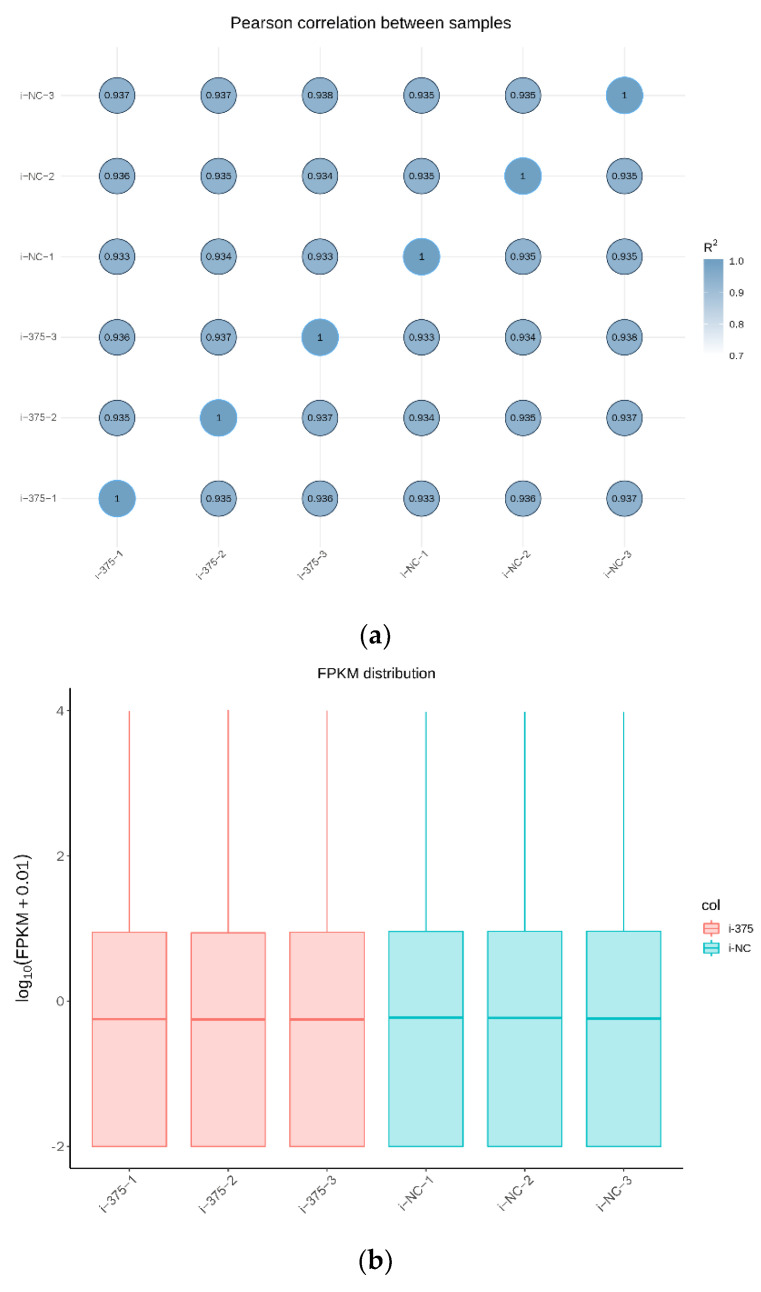
The correlation between samples and overall expression of mRNA in MAC-T. (**a**) Pearson’s correlation analysis between samples. (**b**) Box-plot diagram of the FPKM of each sample.

The expression levels of the overall genes in each sample are presented in Table S2. The results of Pearson’s correlation analysis between samples revealed that the gene expression patterns were highly correlated among the samples (Figure 2a), further validating the reliability of the experimental design. Further analysis showed that the distribution of FPKM in each block diagram was similar, indicating that the overall gene expression abundance in each sample was similar (Figure 2b).

### 3.4. The Differently Expressed mRNAs in MAC-T after MiR-375 Inhibition

**Figure 3:**
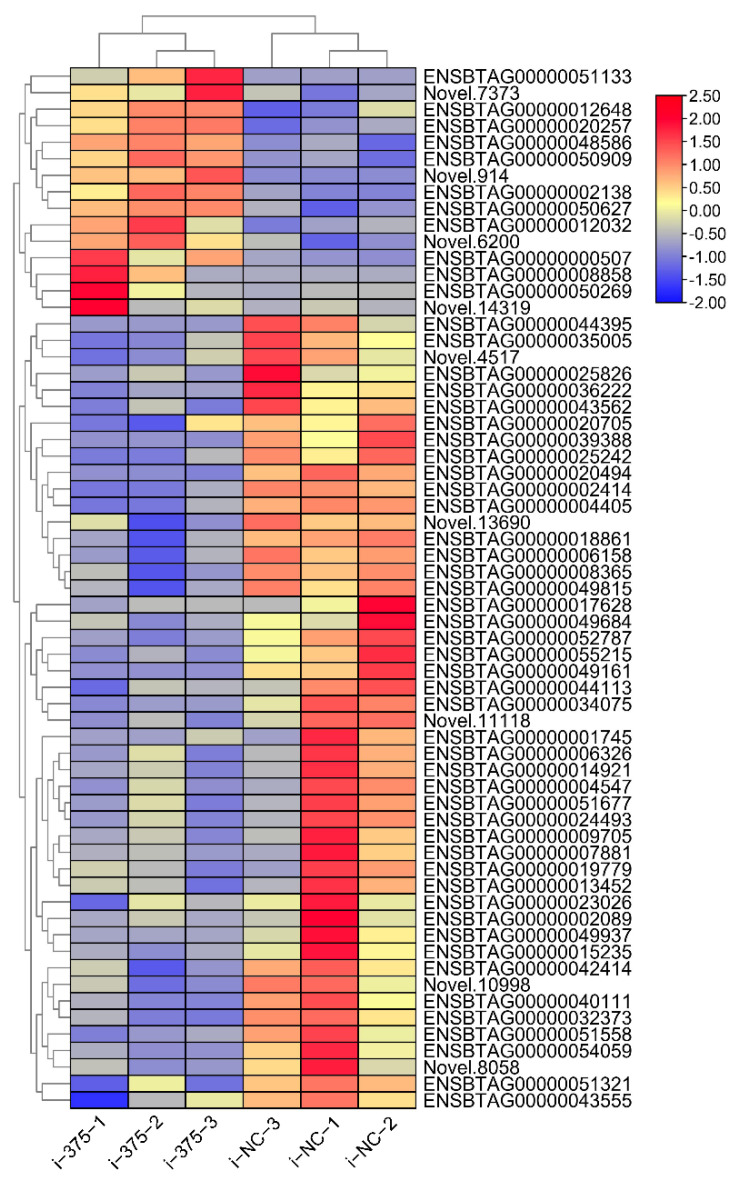
Cluster heat map of DIE-mRNAs between the miR-375 inhibitor and inhibitor NC groups.

Inhibition of miR-375 dysregulated the expression of 63 mRNAs. A total of 48 mRNAs were significantly down-regulated, whereas the remaining 15 were significantly overexpressed (Table S3). We found that inhibition of miR-375 promotes expression of nuclear receptor subfamily 4 group A member 1 (*NR4A1*) and protein tyrosine phosphatase non-receptor type 5 (*PTPN5*). Cluster analysis showed that the high and low expressed genes in the samples were clustered together, indicating that DIE-mRNAs regulate critical processes in bMECs (Figure 3).

### 3.5. Validation of the DIE-mRNAs Using qPCR

**Figure 4:**
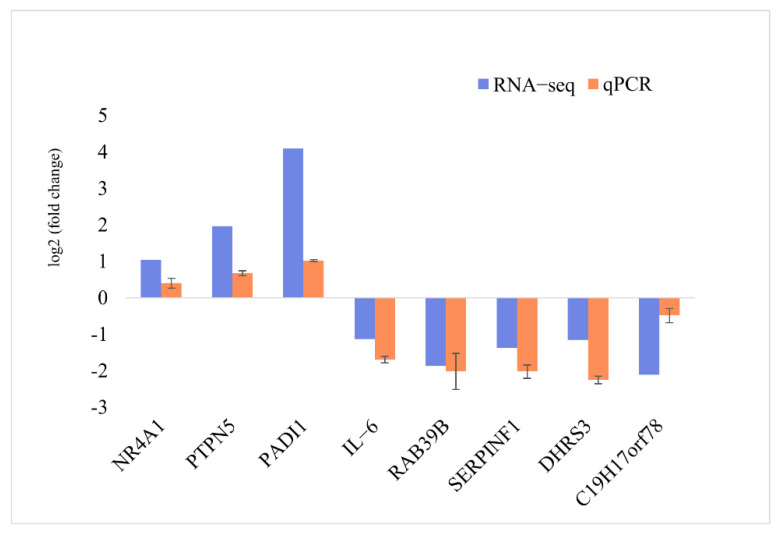
qPCR validation of the DIE-mRNAs identified by RNA-seq analysis.

The expression pattern of eight DIE-mRNAs (three up-regulated and five down-regulated genes) based on RNA-seq was validated using qPCR analysis (Figure 4). The qPCR results were consistent with the RNA-seq results, confirming the accuracy and reliability of the RNA-seq results.

### 3.6. GO Enrichment and KEGG Analyses of the DIE-mRNAs

**Figure 5:**
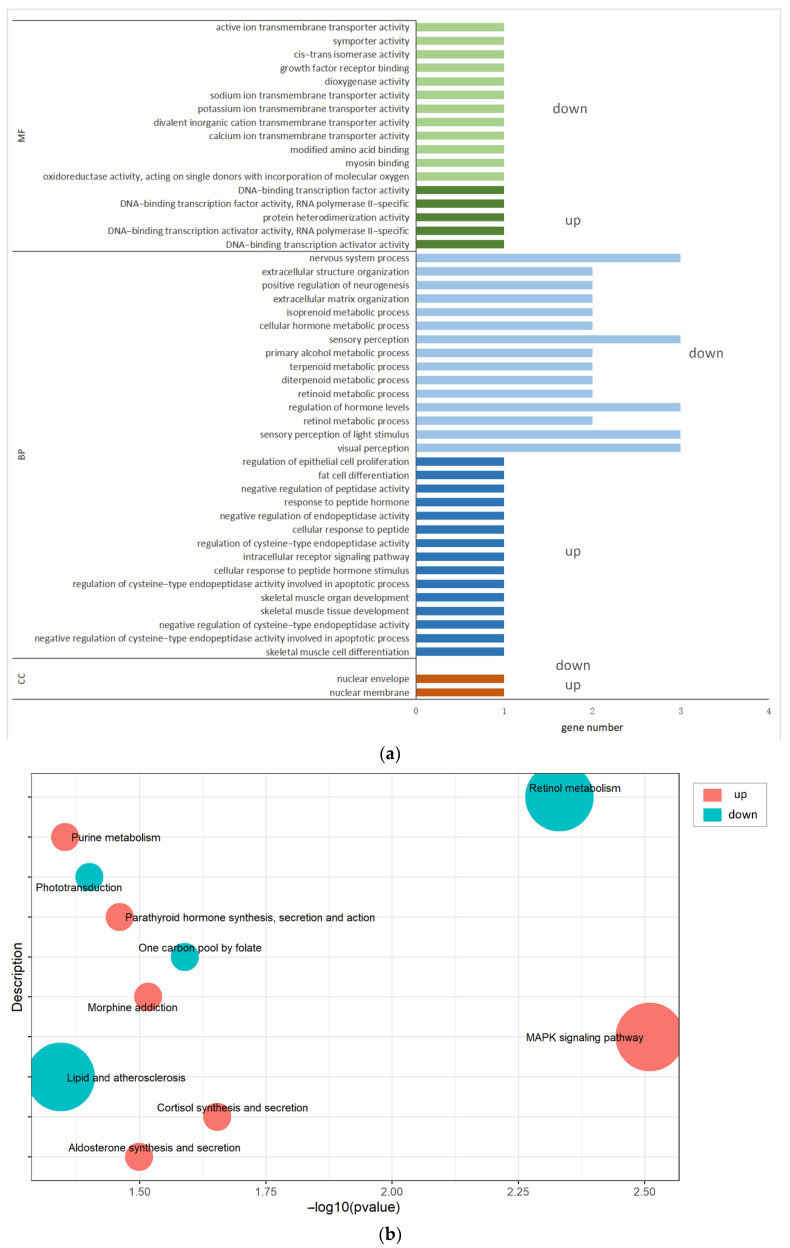
GO enrichment and KEGG signaling pathway analysis of the DIE-mRNAs in bMECs after miR-375 silencing. (**a**) GO enrichment of the DIE-mRNAs in bMECs (*p* < 0.05). (**b**) KEGG enrichment analysis of the DIE-mRNAs in bMECs (*p* < 0.05).

The 63 DIE-mRNAs were annotated to molecular function (MF), biological process (BP), and cellular components (CC) through GO enrichment analysis (Table S4). These DIE-mRNAs enriched nuclear membrane and nuclear envelope under CC. For MF, the DIE-mRNAs enriched DNA-binding transcription activator activity, DNA-binding transcription factor activity, and growth factor receptor binding. However, the enriched BP included regulation of epithelial cell proliferation, intracellular receptor signaling, and extracellular structure organization (Figure 5a). Meanwhile, the KEGG pathway enrichment analysis revealed that the DIE-mRNAs mainly enriched the mitogen-activated protein kinase (MAPK) signaling pathway, retinol metabolism, and cortisol synthesis and secretion (Table S5 and Figure 5b).

## 4. Discussion

This study found that silencing miR-375 down-regulated and up-regulated the expression of 48 and 15 mRNAs, respectively, in MAC-T cells. Regulation of miRNA is a complex process that affects gene expression in the entire cell. MiRNA is directly regulated for some genes, and indirectly regulated for a large number of genes [9]. Therefore, this may be the reason why the down-regulated genes were more abundant than the up-regulated ones after silencing of miR-375. GO revealed that the 63 DIE-mRNAs regulated several biological processes, including regulation of epithelial cell proliferation, the intracellular receptor signaling pathway, and extracellular structure organization. KEGG enrichment analysis further revealed that the DIE-mRNAs regulate the MAPK signaling pathway, retinol metabolism, and cortisol synthesis and secretion. The MAPK signaling pathway regulates the growth and differentiation of cells, adaptation to environmental stress, inflammation, and other important cellular physiological and pathological processes [30,31,32]. In addition, the MAPK signaling pathway participates in the occurrence of mastitis in dairy cows [33]. Other signaling pathways were not closely related to the function of bMECs.

KEGG analysis further revealed that inhibiting miR-375 up-regulated the expression of *PTPN5* and *NR4A1* genes, all regulated via the MAPK signaling pathway. NR4A1 (also called Nur77) is a member of the orphan nuclear receptor family 4A (NR4A), which regulates inflammation and immunity [34,35]. The anti-inflammatory gene *NR4A1* is rapidly expressed in the early stages of inflammatory upon entry of stimuli such as lipopolysaccharide (LPS), or secretion of cytokines such as interleukin-1β (IL-1β), and tumor necrosis factor α (TNF-α) [36]. It indicated that NR4A1 might be an important mediator in the early inflammation. NF-κB plays a critical role in regulating inflammation [37,38]. In vascular endothelial cells, NR4A1 up-regulates IκBα expression but inhibits the activation of NF-κB by binding to the IκBα promoter [39]. NR4A1 modulates the expression of NF-κB by directly interacting and blocking the binding of p65 to its κB, inhibiting the secretion of pro-inflammatory cytokines [40]. Modulating NR4A1 expression induces NF-κB dependent activation of macrophages [41]. The interaction between NR4A1 and NF-κB/p65 in microglia alleviates brain injury caused by cerebral ischemia, thus inhibiting neurogenic inflammation [42]. NR4A1 inhibited LPS-induced inflammation in acute liver injury by directly binding to TRAF6 [43]. Therefore, NR4A1 may be a potential target for regulating and preventing inflammation in bMECs. Meanwhile, miR-375 modulates mastitis in dairy cows by regulating the expression of *NR4A1*.

Reversible phosphorylation of tyrosine residues plays a key role in many signaling pathways [44], catalyzed by protein tyrosine phosphatases (PTPs) [45]. In humans, the *PTP* genes have been linked to several diseases and, thus, potential therapeutic targets in such complications [46]. *PTP* genes are divided into four families. The non-receptor protein tyrosine phosphatase (PTPN) belongs to the class I family [47]. The *PTPN* gene family members regulate numerous physiological processes and participate in the development and pathogenesis of numerous diseases [47,48,49]. PTPN5 (also known as striatum-enriched protein tyrosine phosphatase (STEP)) mainly participates in regulating neuronal signal transduction, and abnormal expression of this protein impairs motor control and cognitive function [50,51]. PTPN5 binds and reduces the affinity to MAPK substrates, negatively regulating the activity and cell localization of MAPK family members. These events block the kinase nuclear translocation of some cellular functions, such as inflammation [52]. In vivo studies revealed that PTPN5 inhibits the growth of breast tumors by blocking the epidermal growth factor (EGF)-induced MAPK signaling pathway [53]. Additionally, high PTPN5 activity decreases with aging [54], whereas PTPN5 deficiency induces neuronal inflammation and exacerbates ischemic brain injury [55]. Inhibition of PTPN5 reverses cognitive deficit impairment in mouse models with Alzheimer’s disease [56]. Our RNA-seq results revealed that PTPN5 modulates inflammation by inhibiting the MAPK signaling pathway in the mammary gland of dairy cows with mastitis. Therefore, silencing miR-375 alleviates mastitis in cows by promoting *PTPN5* expression and while inhibiting the MAPK signaling pathway.

It is worth noting that IL-6 is a key inflammatory cytokine. The infected mammary glands can promote the secretion of IL-6 and initiate the inflammatory response and body immunity by activating various signaling pathways [57]. It has been reported that the expression of IL-6 is positively correlated with the severity of mastitis in dairy cows [57,58]. We found that inhibition of miR-375 down-regulated *IL-6* expression. Therefore, the down-regulation of IL-6 in the miR-375 inhibition group suggested that inhibition of miR-375 might alleviate the inflammatory response.

## 5. Conclusions

In summary, miR-375 silencing dysregulated the expression of 63 mRNAs in bMECs. Additionally, miR-375 silencing increased the expression of *NR4A1* and *PTPN5* genes, all anti-inflammatory genes, via the MAPK signaling pathway. Given silencing of miR-375 significantly up-regulates *NR4A1* and *PTPN5* gene expression, miR-375 potentially promotes inflammation in the mammary gland through the MAPK signaling pathway. The findings of this study provide a new perspective on treating mastitis in cows.

## Figures and Tables

**Table 1:** qRT-PCR primers.

Gene Name	Primer Sequence (5′-3′)	Product Length/bp
miR-375	F: CTCTGCTTTTGTTCGTTCGG	64
	R: AGTGCAGGGTCCGAGGTATT	
*NR4A1*	F: CGGCTTTGCTGAACTGTCTC	80
	R: CCAGACGGAGGATAAAGAGC	
*PTPN5*	F: AGGGCTTCGGCTATCTCAT	228
	R: TGTGAGGGTTGGGAAGGAT	
*PADI1*	F: ACCTGTCCTACGCAGTGGC	139
	R: TCAGGCAGGGTCTTGGTG	
*IL-6*	F: CACTCCATTCGCTGTCT	227
	R: GTGTCTCCTTGCTGCTT	
*RAB39B*	F: TTACCAACCGCAGGTCTTTCC	164
	R: ATACGCAGCAGCCAGTTTCTC	
*SERPINF1*	F: CGCCAATGTGCTGCTGTCT	135
	R: CCGTGGATGTCTGGGTTACTG	
*DHRS3*	F: AATGCCTGAAGGAGACGACG	224
	R: GGTGTTGATGTGCTGGGACTT	
*C19H17orf78*	F: TGGTTCTGGGAGCAAAGTGA	271
	R: AGGAGTTACGGAGGTAGTAGTGG	
*GADPH*	F: GGCATCGTGGAGGGACTTATG	186
	R: GCCAGTGAGCTTCCCGTTGAG	
*RPS18*	F: GTGGTGTTGAGGAAAGCAGACA	79
	R: TGATCACACGTTCCACCTCATC	

**Table 2:** Quality assessment and comparison of sequencing data with reference genomes.

Group	Sample	Raw Reads	Error (%)	Q30 (%)	GC (%)	Clean Reads	Clean Bases	Total Mapped
Inhibitor group	i-375-1	43,314,930	0.03	93.24	47.38	43,038,262	5.95G	41,300,139 (95.96%)
	i-375-2	42,091,914	0.03	92.55	47.27	41,737,124	5.77G	39,883,875 (95.56%)
	i-375-3	46,367,836	0.03	93.29	47.23	46,051,060	6.37G	44,258,208 (96.11%)
Inhibitor NC group	i-NC-1	41,433,410	0.03	93.02	47.07	41,141,648	5.7G	39,472,120 (95.94%)
	i-NC-2	46,829,314	0.03	92.6	47.08	46,481,256	6.42G	44,516,188 (95.77%)
	i-NC-3	46,103,512	0.03	93.28	47.05	45,797,992	6.33G	44,030,956 (96.14%)

## Data Availability

The RNA-seq data (GSE197498) were uploaded to GEO (https://www.ncbi.nlm.nih.gov/geo/query/acc.cgi?acc=GSE197498 (accessed on 26 February 2022)).

## Supplementary Materials

The following supporting information can be downloaded at: https://www.mdpi.com/article/10.3390/ani12111431/s1, Table S1: The list of RNA quality in the miR-375 inhibitor and inhibitor NC groups; Table S2: The list of FPKM for each gene in miR-375 inhibitor and inhibitor NC group; Table S3: The list of DIE-mRNAs in the miR-375 inhibitor vs. inhibitor NC groups; Table S4: The list of GO enrichment analysis results for DIE-mRNAs; Table S5: The list of KEGG enrichment analysis results for DIE-mRNAs.
